# The implementation potential of a method to monitor empirically-supported children’s mental health treatment through claims data

**DOI:** 10.1186/s12913-021-07317-z

**Published:** 2021-12-18

**Authors:** Sarah Cusworth Walker, Noah Gubner, Aniyar Iztguttinov, Felix Rodriguez, Paul Davis, Aaron Lyon, Suzanne Kerns, Eric Bruns, Jiage Qian, Georganna Sedlar

**Affiliations:** 1 Department of Psychiatry and Behavioral Sciences, University of Washington School of Medicine, 1959 NE Pacific St, Seattle, WA 98195 USA; 2 Washington State Health Care Authority, P.O. Box 45330, Olympia, WA 98504-5330 USA; 3 Department of Psychiatry and Behavioral Sciences, University of Washington School of Medicine, 2012 Skagit Lane, Miller Sall, Box 353600, Seattle, WA 98195 USA; 4 School of Social Work, University of Denver, Craig Hall, 2148 South High, St. Denver, CO 80210 USA; 5 Department of Psychiatry and Behavioral Sciences, University of Washington School of Medicine, 6200 NE 74th Street, Building 29, Suite 110, Seattle, WA 98115 USA; 6 Johns Hopkins Bloomberg School of Public Health, 615 N Wolfe St, Baltimore, MD 21205 USA

**Keywords:** Children’s mental health, Evidence-supported treatment, Evidence-based treatment, Pragmatic measures, Quality measures, State systems

## Abstract

**Background:**

The delivery of evidence-supported treatments (EST) in children’s mental health could be a valuable measure for monitoring mental healthcare quality; however, efforts to monitor the use of EST in real world systems are hindered by the lack of pragmatic methods. This mixed methods study examined the implementation and agency response rate of a pragmatic, claims-based measure of EST designed to be applied as a universal quality measure for child psychotherapy encounters in a state Medicaid system.

**Methods:**

Implementation potential of the EST measure was assessed with healthcare leader rankings of the reporting method’s acceptability, appropriateness and feasibility (*n* = 53), and post-implementation ratings of EST rate accuracy. Ability of the healthcare system to monitor EST through claims was measured by examining the agency responsivity in using the claims-based measure across 98 Medicaid-contracted community mental health (CMH) agencies in Washington State.

**Results:**

The analysis found the reporting method had high implementation potential. The method was able to measure the use of an EST for 83% of children covered by Medicaid with 58% CMH agencies reporting > 0 ESTs in one quarter. Qualitative analyses revealed that the most significant barrier to reporting ESTs was the operability of electronic health record systems and agencies’ mixed views regarding the accuracy and benefits of reporting.

**Conclusions:**

Measurement of child mental health ESTs through Medicaid claims reporting has acceptable implementation potential and promising real world responsiveness from CMH agencies in one state. Variation in reporting by agency site and low to moderate perceived value by agency leaders suggests the need for additional implementation supports for wider uptake.

## Background

There is considerable interest in building the data capacity of state mental health systems to monitor service quality. State policymakers view better mental health quality measurement as critical, with nearly half of all states (43%) indicating that mental health treatment monitoring is a top priority for improved healthcare delivery [1]. The Institute of Medicine (IOM) identified quality measurement as a key strategy for improving mental and substance-use conditions [2], and recent investments through Medicaid and Children’s Health Insurance Programs [3] are supporting the testing of multiple quality metrics in children’s mental healthcare [4–6].

A sizeable gap in current approaches to quality measurement for children’s mental health services is the lack of a method for measuring the use of evidence-supported treatment (EST), despite ESTs being recommended as the first line treatment for most childhood mental health disorders [7]. This limitation is repeatedly noted by scholars focused on developing and testing mental health quality metrics at the system level [8, 9]. ESTs are missing from national registries of quality measures such as National Quality Forum and Children’s Health Insurance Programs (CHIP) because implementation feasibility is a priority for measurement selection at this level. In order to obtain recommended status, measures must demonstrate reliability and validity in administratively-collected (rather than investigator-collected) data (NQF measure testing criteria, August 2014). No healthcare system has yet captured EST at the volume needed to conduct these psychometrics. Alongside other measurement points, such as time to follow up [10], appropriate psychotropic prescription [11], and use of client feedback measures to guide treatment [12–15], the ability to routinely measure ESTs would significantly improve a system’s capacity to measure and support quality services in treating mental health needs.

Billing claims are a common data source for obtaining quality measures in healthcare [4, 5]. Claims are reports sent by a healthcare provider organization to a payer (insurance company or state, depending on the system) to obtain reimbursement or to confirm the costs of services. The pattern and types of services are extracted from this data to determine whether clients received optimal care. In children’s mental health care, for example, claims-data has been used to determine whether children’s released from inpatient psychiatric care received a follow up visit within 30 days, per best practices [6]. A key advantage of claims data is that it provides universal data, i.e., all clinics, within a state healthcare system. Universal data on quality lends itself to a number of potential applications to encourage higher value care, including routine performance feedback, alternative payment models (APM), and real world health services research [8, 14]. Obtaining information about ESTs through claims-based data would improve the precision of these real world strategies to monitor the quality of children’s mental healthcare. To date, there are no studies of the implementation potential of a claims-based reporting method of EST across a statewide system.

### Monitoring EST delivery

Implementing a universal measure of EST use through claims data faces at least two considerable challenges. The first is decoupling the measurement of a clinician’s use of an EST from a state’s investment in high cost training and consulting efforts. The second is engaging the motivation and ability of providers to use the EST measure accurately. We discuss recent innovations reported in the treatment and implementation literature, particularly in the elucidation of core elements of effective treatments that offer opportunities to overcome these challenges.

Current efforts to monitor EST in real world systems is limited to a handful of treatments because clinical language among EST protocols are idiosyncratic and thus require specialists to determine whether clinicians are delivering models to the specifications of treatment developers [16–22]. For example, in Sedlar et al.’s [16] case study of a unified fidelity system for four different ESTs (Incredible Years, Parent Child Interaction Therapy, Homebuilders), the quality assessment of each was monitored by a trained specialist who then translated individual program fidelity ratings into a standard score the state used to monitor provider quality. Similarly, the statewide effort to support evidence-based prevention services across Pennsylvania began with the aim to support any eligible evidence-based prevention program, but quickly narrowed to a handful of programs in order for state-supported specialists to sufficiently absorb the specific protocols used by each program [18].

States gain some advantages in investing in just a handful of training and consultation purveyors [21] particularly in the economies of scale [23], and supporting workforce training is an important role for state mental health agencies. At the same time, therapists increasingly receive evidence-supported training through graduate schools or other workforce development efforts. Without a measure to track these ESTs, statewide EST counts will be underestimated and lose value as a universal measure. Further, it is infeasible for a state to independently verify the competence of all clinicians reporting a wide variety of ESTs using typical adherence-monitoring methods (e.g., video or audio review, feedback, coaching) given the costs and resource burden of this approach. To ensure that quality monitoring at scale is decoupled from whatever training and consultation support the healthcare system may also be purchasing, the system needs a clear definition of evidence-supported psychotherapy that can feasibly encompass the hundreds of unique treatment protocols meeting the evidence-supported designation [24].

The pragmatic monitoring of ESTs is increasingly moving away from defining quality as the implementation of therapeutic techniques in an orderly sequence of sessions according to a set protocol (delivering EST to fidelity), to monitoring the application of evidence supported clinical elements suited to the therapeutic needs of each client [25, 26]. A common elements framework revolutionizes the ability to monitor ESTs at scale because it makes the monitoring of potentially hundreds of protocols more efficient, and it provides a model of treatment (e.g., guided clinician discretion) that can conceptually justify the session-level tracking of EST. The common elements model allows for this by individual protocols within treatment families and translating idiosyncratic clinical language within specific protocols to standard terms (e.g., “trauma narrative” within TF-CBT protocol/model becomes “exposure”). This conceptually grounds the ability to capture EST as a universal measure, and makes it at least theoretically possible to conduct medical record reviews to confirm the delivery of EST per session as long as therapists use standard terminology in documenting delivered services.

The second significant challenge faced by a traditional model of EST fidelity monitoring is the infrequency with which therapists are able to complete a full course of EST-based treatment in community-based settings. Children covered through managed care plans receive an average of only 4.6 sessions [27], and a study by Gopalan et al. [28] showed that 40-60% of children who receive outpatient mental health treatment drop out quickly. Consequently, the baseline for completing EST manuals in Medicaid or managed healthcare is likely to be so low as to be unusable for quality monitoring purposes. Rather than monitoring full protocols, health care systems need a process for tracking the use of EST components in individual sessions. The common elements approach provides conceptual grounding for thinking about evidence-supported psychotherapy as the delivery of an effective clinical technique, appropriate to the client’s presenting need, within a single session and in the context of a treatment plan that clearly lays out an evidence-informed approach [29].

### National quality measures

Efforts to assess the feasibility of quality measures in child mental health treatment have typically involved gathering expert and stakeholder feedback during measure identification and development [6, 30], and then testing the measures in real world healthcare data systems. A component of validation involves demonstrating that the claim-based measures are truly universal and identify an adequate representation of clients or agencies in real world data systems [6, 31, 32]. Research on the accuracy of administrative measures in behavioral health is still very much in progress and the extant literature suggests claims-based quality measures currently identified for child mental health treatment have low adherence. For example, less than 30% of eligible cases meet quality standards as defined by follow up visits, depression screening, and polypharmacy regulations [6]. As thereare no studies examining EST adherence using claims-based measures, there are no available benchmarks to use to compare the feasibility of various EST measurement systems. In a case study of claims-reported EST in Los Angeles County [33], the authors present a 25% EST reporting rate for a single EST (Mapping and Adapting Practice) as reflective of high reporting. Given the lack of research in this area, the ability of an EST measurement method to identify EST use for over 20-30% of child/adolescent cases would appear to exceed available benchmarks.

### Study context

The development of claims-based EST reporting for child mental health services in Washington State, USA aligns with national approaches to develop quality measures, including the review of the evidence literature and integration of feedback from experts and stakeholders [30]. EST tracking in Washington State began in response to a state legislative mandate (2012 HB2536, RCW.43.20C.020) to calculate financial investments in evidence- and research-based practices as a proportion of all service costs across three child-serving systems statewide (mental health, juvenile justice, child welfare). To track EST investments within mental health services, the state directed Medicaid-contracted mental health agencies to report the use of an EST in claims forms (2300 form) as a preauthorized service attached to a billing code for an eligible psychotherapy service (CPT code).

Washington State’s approach was developed to allow EST use to be fully verified through medical record review of session-level encounters and treatment plans. This verification is made possible by an annually updated manual for reporting EST, as well as guidance for practitioners’ documenting ESTs [29]. The manual includes a listing of unique codes that correspond to various ESTs for children and adolescents. This method of tracking and verification is consistent with tracking approaches for other child mental health quality measures, particularly those that reflect psychosocial services (e.g., ADHD follow up visits). The guidance for session level documentation is based on well-established common clinical elements found across ESTs for various diagnostic categories (e.g., disruptive behavior, anxiety, traumatic stress [26, 34, 35];) Therapists document the EST in the treatment plan and in claims reporting (e.g., CBT for Anxiety) and also document the delivered clinical elements in the progress notes of individual sessions (e.g., cognitive coping, psychoeducation for child). While the EST treatment family or specific EST protocol gets reported through claims (e.g., Trauma-Focused CBT), the clinical elements (e.g., exposure) are currently documented only in treatment plans and progress notes and are not captured through this method of reporting (Table 1).

**Table 1:** Example of the treatment family “CBT for depression”: eligible trainers and common elements

Trainers (not exhaustive)	Modifier code (reported in claims)	Essential elements (reported in treatment plan)	Common elements^a^ (reported in progress notes)
Modularized Approach to Therapy for Children with Anxiety, Depression, Trauma, or Conduct Problems (MATCH-ADTC)	085	Behavioral activation	[essential elements] Psychoeducation for child
Primary and Secondary Control Enhancement Trainig (PASCET)	209	Problem solving	Psychoeducation for caregiver
Managing and Adapting Practice (MAP)	153	Cognitive restructuring	Mood monitoring
Harborview CBT+ Learning Collaborative	153		Goal setting

^a^The common elements list is not exhaustive. Essential elements and common elements are reported in therapistservice plans and notes, only the training organization modifier code is reported in claims

The process for identifying the common clinical elements of ESTs is scientifically informed and thus similar to the first stage of quality measurement development used by national efforts [4, 5]. The ESTs eligible for reporting are updated annually, incorporating information from systematic reviews and clinical guideline reviews released from the American Psychiatric Association, the American Psychology Association and National Institute for Health and Care Excellence. Only interventions meeting the evidence standards of Level 1 (systematic reviews and meta-analysis of randomized controlled trials), Level 2 (one or more randomized controlled trials), or Level 3 (controlled trial, no randomization) from APA-sponsored reviews [26, 36, 37] are added to the unique EST claims codes each year.

In the remainder of this paper, we present mixed methods data on the implementation potential of this particular reporting method of capturing EST-linked claims across all Medicaid services in a state healthcare system. Methods were informed by the approaches used by pediatric healthcare Centers of Excellence to develop and validate quality metrics [4], and other studies of the performance of quality measures from claims-derived data [38]. Specifically, we hypothesized that the method of documentation and reporting of EST through claims would be rated as acceptable, appropriate and feasible by mental healthcare leaders and providers prior to implementation of the reporting method, and rated by leaders as an accurate measure of EST use following implementation. We did not expect to see meaningful differences on these outcomes by job type (managers vs. therapists). We also examined the percent of provider agencies who reported any EST (defined as “reporting agencies”) as a proportion of all agencies, as well as the range of reported EST among reporting agencies. Without established benchmarks for either metric (provider agencies who report any EST, and the range of EST reporting), we interpret the findings in light of the 30% adherence rate of other behavioral healthcare quality metrics. We also report qualitative feedback on EST reporting to guide future improvement of the reporting system.

## Methods

### Procedures and participants

The study relied on two different sources of data to assess the implementation potential and use of the EST reporting method. The implementation potential and perceived accuracy of the reporting method was captured through surveys sent to community mental health leaders prior and after the reporting method was introduced. Real world use of the reporting method was examined with claims data from the Washington State Health Care Authority Medicaid Management Information System (MMIS) to assess the distribution of reporting across all community mental health agencies in the state. Participants in the study included community mental health directors, clinical and quality assurance managers, clinical supervisors, and frontline therapists in Washington State.

#### Implementation potential and perceived accuracy

Implementation potential and perceived accuracy was assessed with two surveys (pre-implementation and post-implementation of the reporting method) sent to clinical directors, supervisors, quality assurance managers, and clinicians. In 2017, the research team conducted ten, 1.5 h, regional workshops in person or through a video link to all 98 Medicaid-contracted mental health agencies across Washington State. The workshops involved didactics, case examples, and discussion to provide information about the method for reporting ESTs using a common elements framework. Following each workshop, participants were administered a survey regarding the implementation potential of the EST reporting method [39].

**Fig. 1:**
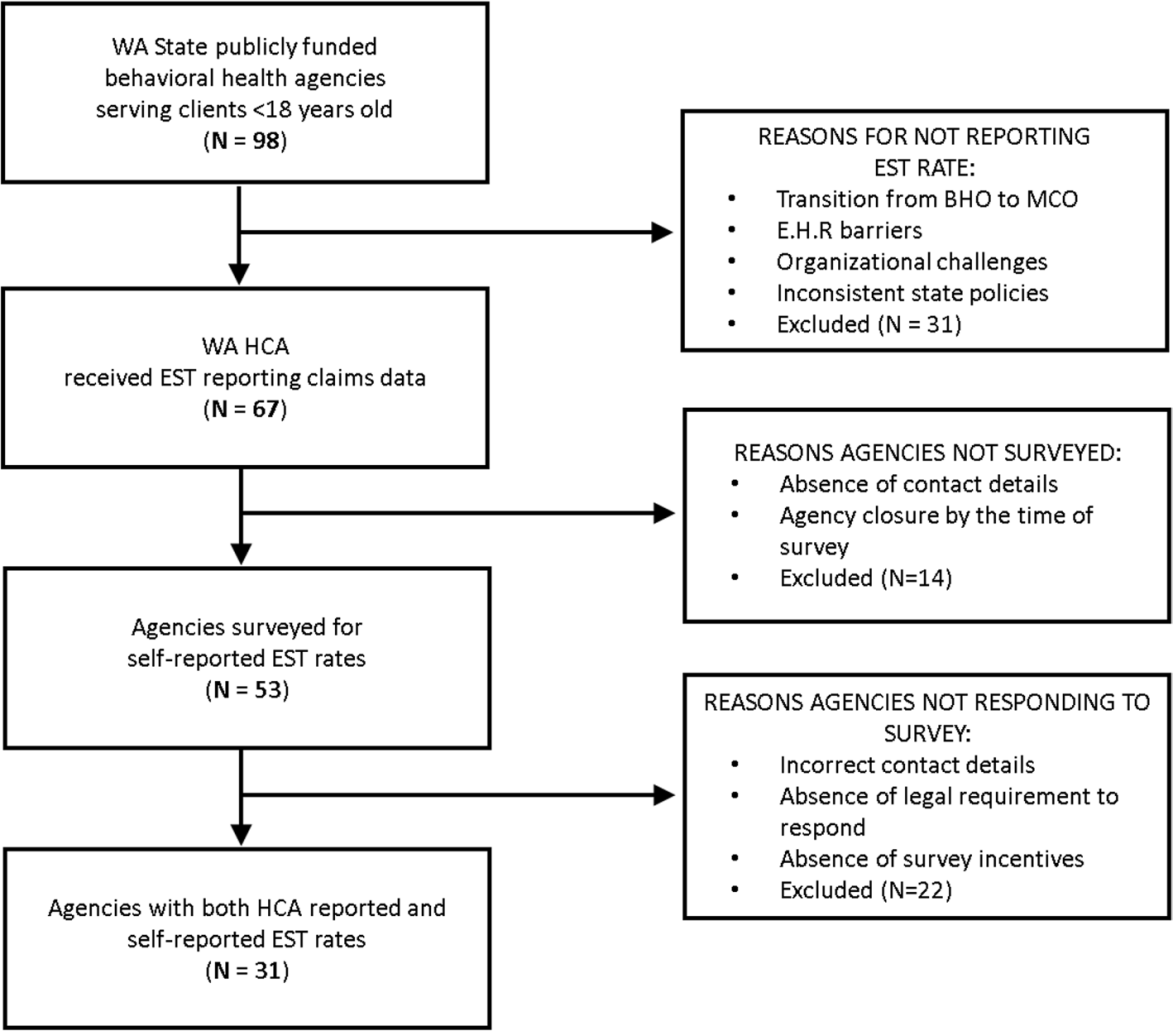
Consort Diagram of EST-Reporting Agencies and Survey Response

Following statewide roll out, surveys were again administered to Medicaid-contracted agencies to report on the ability to report, and the accuracy of a state claims-derived rate of agency-level EST. Surveys were sent to clinical directors and agency quality leads from contact lists populated from multiple sources of data (state listservs, websites, and contact lists managed by the research team) representing all community mental health agencies that reported at least one child mental health encounter in the second quarter of Fiscal Year 2018 (*n* = 53 agencies). Research staff sent up to three emails, and called agencies up to three times before denoting the agency as nonresponsive (Fig. 1).

#### Agency use of the EST measure

The use of the EST reporting method was examined by comparing agencies that reported at least one EST-linked claim within and across geographic regions in the state. This was derived from claims data reported from provider agencies to the state Health Care Authority (HCA) through the MMIS. Claims data were captured in the second quarter of State Fiscal Year (SFY) 2018, and aligned with distribution of the post-implementation survey. EST rate within reporting agencies was compiled and calculated by analysts within the HCA using an algorithm jointly developed between the research team and researchers at HCA.

### Measures

#### Implementation potential

Pre-implementation implementation potential was assessed with the short form (12-item) version of three scales of implementability [39]. Collectively, the Acceptability of Intervention Measure (AIM), Intervention Appropriateness Measure (IAM), and Feasibility of Intervention Measure (FIM) includes 12 items that monitor and evaluate the success of innovation implementation efforts. Each statement is rated using a Likert scale with response options ranging from 1 to 5, with 1 referring to “complete disagreement” and 5 indicating “complete agreement”. The developers of the tool were previously able to demonstrate content validity (Cronbach alphas of 0.85 to 0.91) and test-retest reliability (coefficients of 0.73 to 0.88) of the three measures [39]. Two open-ended questions were added to the survey to solicit feedback on the reporting method, “what was helpful about the [reporting method],” and “what would you change about the [reporting method].”

#### Perceived accuracy

Post-implementation assessment of EST reported accuracy was assessed with a four-item survey administered to individual mental health service agencies via email or through a phone call. Respondents were asked to rate the accuracy of the state reported EST rates for the period of April through June 2018 (Quarter 2). Providers rated accuracy for two dimensions: 1) Accuracy compared to the agency’s reported rate of EST services as submitted through claims data; 2) Accuracy compared to the agency’s delivery of all ESTs, including those not reported through claims data. The first two questions in the survey asked the respondent to rate the accuracy of the state reported rate for their agency from 0 to 3 (not well, a little well, very well), and respondents were referred back to the EST common elements reporting guidance as a basis for their responses: *How well does the reported EST rate match your agency’s reported use of evidence-based practices? How well does the reported EST rate match your agency’s actual use of evidence-based practices?* The third question asked the agency to estimate their agency’s actual range of EST use for children’s mental health services (0-10%, 11-30%, 31-50%, 51-70%, 71-100%). Respondents were also asked to respond to open-ended questions about the barriers experienced in EST reporting. Thirty-one of 53 contacted agencies responded to the survey assessing post-implementation perceived accuracy of claims-derived rates. This represents 65% of the sampled agencies (*n* = 53) and 32% of the total number of Medicaid contracted agencies in the state (*n* = 98). Seven of the ten administrative regions were represented in agency responses. We did not collect data on agency characteristics that would allow for assessing predictors of response/nonresponse to the survey.

#### Agency use of the EST measure

Use of an EST service in an individual psychotherapy session is reported using a three-digit code (in this case, a Current Procedural Terminology (CPT) code for psychotherapy (group, individual, family). These codes are applied for any psychotherapy service that exceeds 30 min. The agency level of EST is calculated as the rate of EST services with CPT codes to all eligible psychotherapy services, where the numerator is the number of eligible sessions with an EST coded for a session, and the denominator is the count of all eligible psychotherapy sessions (group, individual, family). Rate of EST delivery excludes all non-psychotherapy encounters billed under mental health services (e.g., medication management, case coordination, brief consultations, peer services). EST rates were calculated for 67 mental health agencies across eight regions in the state. Two regions did not have data available for analysis as they had recently transitioned from a regional behavioral health managed care infrastructure to managed care organization (MCO) contracts and data systems for reporting services were in transition.

### Data analysis

#### Implementation potential

Reliability analyses were used to confirm factor structure of the pre-implementation measure [39]. One-way analysis of variance (ANOVA) models were used to assess whether perceived feasibility varied by respondent job title. Content analysis was used to interpret responses to open-ended questions included in the survey. Content analysis employed open-coding to derive themes emerging from responses [40]. To enhance reliability, responses were independently coded by three team members who met to discuss and refine the coding scheme. The responses were then reviewed and recoded by one coder, when needed, to be consistent with the final codes and themes and final codes were confirmed by all three team members through consensus.

#### Perceived accuracy

Perceived accuracy at post-implementation was assessed by the percent of agencies responding that state-derived EST rate for their agency corresponded “very well” to the claims submitted by the agency. Given the lack of benchmarks in this area, we considered 30% of agency responses falling within “Very well” as an indication of acceptable adherence to the measure [6]. Paired t-tests were used to assess mean differences in the state reported EST rate vs. the agency’s estimated actual use of ESTs in clinical practice. Responses to open-ended questions about barriers to use were coded followed the same qualitative coding procedures described previously, using consensus coding methods using multiple coders to determine themes [40].

#### .Agency use of the EST measure

Descriptive statistics were used to assess the proportion of agencies reporting any EST and the percent coverage of all youth in public mental health services falling within reporting agencies. We validated the reported rates by region using data from a previous study that gathered information about EST use through surveys filled out by agency leaders [41]. A low response rate (e.g., EST reporting not exceeding 5% in any agency in that region) was considered problematic as previous studies in Washington State indicated that all regions had EST use that exceeded 5% when aggregated across CMH agencies in that region [41].

## Results

### Implementation potential

#### Acceptability, appropriateness, feasibility

Of the 98 Medicaid-contracted agencies in the state, participants from 47 agencies responded to the workshop survey assessing the implementation potential of the EST tracking method (48% response rate, Table 2). Respondents represented all 10 behavioral health administrative regions in the state and included clinical directors/supervisors, *n* = 46; clinicians, *n* = 6; administrators/quality assurance, *n* = 23. Reliability was strong across all three scales as measured by Cronbach alpha, acceptability = 0.94; appropriateness = 0.95; feasibility = 0.95. Only one of the respondents (clinician) rated the EST reporting method as unacceptable on all three scales (rating of 1), and this individual was removed from subsequent group comparison analyses as an outlier.

**Table 2:** Implementation potential of the EST measurement method

Construct	Mean (SD)			
	Total	Administrators	Clinicians	Supervisors
**Acceptability scale score**	**4.06 (0.77)**	**4.34 (0.62)**	**4.00 (0.77)**	**3.93 (0.82)**
The Reporting Guides meet my approval.	4.07 (0.78)			
The Reporting Guides are appealing.	4.00 (0.86)			
I like the Reporting Guides.	4.09 (0.91)			
I welcome the use of the Reporting Guides.	4.07 (0.85)			
**Appropriateness scale score**	**4.01(0.76)**	**4.34 (0.66)**	**3.70 (0.84)**	**3.89 (0.76)**
The Reporting Guides seem fitting.	3.99 (0.85)			
The Reporting Guides seem suitable**.**	3.97 (0.88)			
The Reporting Guides seem applicable.	4.12 (0.78)			
The Reporting Guides seem like a good match	3.95 (0.79)			
**Feasibility scale score**	**4.03 (0.76)**	**4.26 (0.66)**	**3.70 (0.78)**	**3.94 (0.78)**
The Reporting Guides seem implementable.	3.97 (0.91)			
The Reporting Guides seem possible.	4.09 (0.80)			
The Reporting Guides seem doable.	4.11 (0.84)			
The Reporting Guides seem easy to use.	3.96 (0.78)			
**Total implementation scale score**	**4.03 (0.72)**	**4.31 (0.61)**	**3.80 (0.76)**	**3.92 (0.74)**

Responses averaged across the implementation scales indicated high acceptability, appropriateness and feasibility among Administrators with means exceeding a score of 4 (“Agree”) on all scales (acceptability = 4.34; appropriateness = 4.34; feasibility = 4.26). Among Supervisors, mean scores fell just below an average score of 4 (“Agree”) on all scales (acceptability = 3.93; appropriateness = 3.89; feasibility = 3.94). Clinicians rated acceptability as 4.0 (“Agree”), and appropriateness and feasibility just below 4 (appropriateness = 3.70; feasibility = 3.70). Administrators had the highest acceptability scores followed by Supervisors and Clinicians (Table 1). There were no significant differences between groups when comparing scale scores (Acceptability: F_(3)_ = 1.45, ns; Appropriateness: F_(3)_ = 2.32, *p* = 0.08; or Feasibility: F_(3)_ = 1.81, ns).

#### Perceived accuracy

Agencies responding to the accuracy survey had claims-derived EST rates ranging from 0 to 55%, with a mean of 14%. When asked to rate the accuracy of the claims EST rate for their agency, provider responses were polarized with equal numbers judging the accuracy as high or low and slightly fewer agencies reporting the rate as “a little” accurate (“Not well,” *n* = 12, 39%; “A little,” *n* = 7, 23%; “Very well,” *n* = 12, 39%). When asked about the accuracy of the EST rate compared to their judgement of how many ESTs were actually delivered, agencies were more likely to report low accuracy (“not well,” *n* = 20, 65%; “very well”, *n* = 5, 16%; “a little” *n* = 4, 13%). The average assessed accuracy for claims-reported ESTs (M = 2.00, SD = 0.89) was slightly higher than assessed accuracy for ESTs delivered regardless of whether they were reported through claims, (M = 1.48, SD = 0.78), and the difference was statistically significant (t_(28)_ = 3.78; *p* = .001).

#### Agency use of the EST measure

Data obtained from the Healthcare Authority for Quarter 2, SFY 2018 on agency EST rates included information from 67 Medicaid-contracted agencies, indicating that 68% (67/98 total agencies) of the agencies were captured through the claims data for the quarter. Of those agencies with claims data, 58% (39/67) reported a level of EST greater than zero. Agencies reporting greater than zero EST captured 83% of all Medicaid-funded psychotherapy services for children/youth in the state, indicating that the reporting method covered the majority of children/youth enrolled in Medicaid.

The agency-reported rate of EST services between regions ranged from 2.3 to 32.1% with a mean of 15.6%, which matched the distribution of EST reporting obtained from the prior study of EST use obtained through surveys where reported EST ranged from 2 to 23% between regions [42] (Table 3).

**Table 3:** Responsivity to EST reporting through claims data across region and by agency in one quarter

Reporting region	# Agencies in region	Agencies reporting > 0% EST	% of youth in a responsive agency/total agencies^b^	Total number of child mh encounters in responsive agencies^**b**^	% EST claim-linked encounters in responsive agencies^**b**^
Region 1	10	4	75%	8988	15%
Region 2	11	6	93%	5160	12%
Region 3	13	12	100%^a^	25,232	17%
Region 4	5	4	99%	15,152	8%
Region 5	6	3	75%	8560	5%
Region 6	1	1	100%	2330	2%
Region 7	16	8	58%	12,913	32%
Region 8	5	1	67%	3094	20%
**State Totals**	**67**	**39**	**83%**	**81,429**	**16%**

^a^In this region, the one EST-nonresponsive agency only reported 5 psychotherapy encounters for the quarter, consequently, the % of youth covered by responsive agencies for the region rounded to 100%. Responsive = agencies reporting more than 0 ESTs in that quarter

^b^Calculated based on agencies reporting > 0% EST in the quarter

### Qualitative findings

#### Acceptability, appropriateness, feasibility

Qualitative analysis of the open-ended responses from the implementability survey yielded three themes: Organization (11 mentions); Clarity (5 mentions); Reflects real world practice (4 mentions). Most respondents commented on the organization of the reporting instruction with the majority of the comments indicating that the guide for reporting had good flow, examples, and was easy to follow. As noted by one respondent, *“[The method has] well thought out information with examples, feedback and the names that go with all of the acronyms.”* In contrast, only one respondent disagreed that the guidance for reporting was easy to follow, noting that *“The [method] is very long, lots of different sections and sometimes it was hard to follow*. *.*.” *.*

Other respondents reflected on the clarity of the content in the method with positive feedback about how information was presented. As noted by a respondent, *“Clear key elements listed for each EST for documentation purposes.”* Finally, four respondents spoke to the real world applicability of the reporting method’s use of a family treatment and common elements structure. As stated by a respondent, *“[The method] addressed a challenge we all face all of the time – periods of active therapy followed by periods of disengagement/reengagement that disrupts the flow of perfect [EST] implementation. It was helpful to know that there was awareness of this reality and flexibility within the framework.”* Overall, qualitative analysis indicated high to moderate acceptability among agency leaders and clinicians with only one respondent who found the reporting method to be difficult to understand.

#### Perceived accuracy

Qualitative analysis, using consensus coding, of the open-ended response in the survey regarding the accuracy of reporting yielded four themes: Barriers due to the Electronic Health Record (EHR; 14 mentions); Therapist level limitations (mentions = 6); Organizational challenges (mentions = 7); and State policies (mentions = 13). In the words of one respondent:

*“There have been two major challenges in our reporting of [ESTs] through the electronic system. The first is that we have recently purchased a new EHR and have had implementation challenges in reporting various data sets as well as the configuration for coding of clinician use of [ESTs]s. Second has been that our EHR configuration team has been challenged and occupied by the ongoing time and resource demands of the MCO/IMC [Managed Care Organization/Integrated Managed Care] transition.”*

*“Our EHR is not able to capture [EST] codes. IT and Data departments had to develop internal process to capture [EST] services provided and report to the County. This is very time consuming and not easy to make on-going changes. I believe we are not using SERI [Service Encounter Reporting Instructions] codes since making changes are challenging. Basically, it comes down to internal capacity and availability of finances to accurately report [EST] services.”*

Each theme also yielded subthemes. Barriers due to electronic health record (EHR) systems was the most frequently cited challenge. Within this theme, respondents reported difficulties in reporting due to an active EHR transition (5 mentions), existing EHRs not having any functionality for reporting ESTs (2 mentions), and existing EHRs having functionality that was inefficient (7 mentions). While highlighting the issues in standardizing the reporting processes, one agency leader expressed his hope that *“new EHR system will support a more optimized and efficient workflow.”*

## Discussion

The present study reports on the implementation potential and agency willingness to using a claims-based measure of EST delivery designed to capture EST as a universal, quality measure. Our findings reveal that reporting EST through claims had high implementation potential, low to moderate perceived accuracy, and a moderate response rate. Qualitative findings strongly suggest that the primary barrier to EST reporting was difficulty with the operability of EHR systems. Overall, these findings provide preliminary support for claims-based measures of EST delivery as a system-level quality measure.

Our study revealed that a significant barrier in EST reporting was EHR functionality. In most states [23], provider agencies retain discretion in the EHR systems they purchase. While outside of the scope of this paper, anecdotal information from our collaborations with providers and EHR companies suggests considerable variation in the ease with which documentation in a progress note field transfers smoothly to a billing field. A lack of smooth functionality in this process leads to disparities across agencies in the amount of human time needed to review and code EST delivery and subsequent rates of agency reporting. Market pressure from provider agencies and payers will likely be the most potent lever for encouraging the redesign of these systems [42].

EST reporting displayed a large range when plotted across agencies (per agency range of ESTs: 2-32%), and that EST encounter data could be captured from a little more than half of Medicaid agencies (58%). This is in line with the performance of other pediatric quality adherence measures where, on average, 20% of pediatric health providers meet the standard for the measure [4]. We should point out, however, that the definition of adherence from these studies and our definition are not exactly equivalent. In other quality measure studies, adherence is typically tracked by examining the combination of encounters, for example whether a follow up medication management service occurred within 30 days [6]. The EST code used in the present study is closer to the encounter codes used for coordinated care in which the single code represents a set of activities that would need to be separately documented in a medical record [43]. Unfortunately, the extant literature does not include a study that attempts to validate reported multi-activity codes with medical record review, so we are unable to assess the relative performance of the EST code to the accuracy of similar efforts. The self-reported rating of accuracy obtained through our study suggests a modest gap between what is reported through claims, and what is happening at an agency when delivering ESTs; although we also note that the agency survey responses were likely to be inflated due to social desirability bias. A fruitful area of future research will be to assess the drivers contributing accuracy in reporting, as well as independent review of medical records and audio recordings of sessions to confirm adherence.

The measures of implementation potential and perceived accuracy in our study are roughly equivalent to methods used by federally funded efforts to establish quality measures in other areas of pediatric health. For example, the Delphi approach employed by the Center of Excellence on Quality Care Measures for Children with Complex Needs (COE4CCN) included a process to collate ratings from stakeholders representing psychology, psychiatry, families, adolescent medicine, state Medicaid, hospitals, and emergency departments [4, 44]. Stakeholders were instructed to rate each measure on a measure’s feasibility for being captured through medical records as well as being reliable and unbiased (1-3 = not feasible; 4-6 = questionable feasibility; 7-9 = feasible). Only measures consistently rated as feasible were included in the field trial process. Similarly, healthcare staff in Washington State, at different levels of management and practice, were asked to rate the common elements-based, EST reporting approach on its implementation potential and found that potential was rated favorably.

Finally, our study reinforces the importance of attending to multiple levels of influence when attempting to encourage the implementation of a new innovation, in this case a reporting method for ESTs [45, 46]. Key themes from qualitative analyses directly map onto implementation frameworks that highlight the important of policy, organizational, and provider-level barriers. In the current study, implementation strategies were largely limited to policy-level factors with the mandated requirement for EST reporting in legislation and no active reimbursement incentives. Implementation strategies at the organizational and provider level were minimal and limited to informational webinars on how to report ESTs accurately. Organizational technical assistance was available but had to be requested by providers and was infrequently accessed. Increased support at any of these levels would be expected to improve EST reporting through claims data.

### Limitations

This study has a number of limitations. The assessment of implementation potential pre-implementation was limited to respondents who voluntarily attended a workshop on the reporting method. This may have introduced self-selection bias favoring the use of ESTs. The rate of response to the post-implementation agency survey regarding the accuracy of EST use was low. Low response rate suggests that individuals with stronger views about EST reporting may have been more likely to complete the survey. The primary value of data obtained with the post-implementation survey is the description of reporting difficulties and descriptive information about perceived accuracy, and should not be interpreted as generalizable findings regarding the actual accuracy of EST reporting. Establishing the accuracy of EST reporting will need to be assessed with more sophisticated methods of validation, including medical record and expert review of sessions. Finally, EST reporting for this study was conducted in a particular political context in which a state law required the state mental health agency to report EST expenditures to the legislature on an annual basis. The timeframe for this study also coincided with a time of contractual transition from regionally managed entities to managed care organizations. This created uncertainty about whether the state would continue to require or enforce EST reporting. It is unclear how this affected the decision of individual agencies to establish the necessary protocols and infrastructure to report, and, consequently, how that might limit the generalizability of the findings for other healthcare systems.

## Conclusion

There is a strong evidence that delivering ESTs supports mental health recovery in children and youth. However, these approaches are under-represented in national measures of pediatric mental health quality. This disconnect is largely due to challenges with identifying a feasible reporting method. This study reports on the implementation potential of monitoring EST in a statewide system through claims-based reporting. Our study found that the stakeholder-rated implementation potential of this method was high although the perceived accuracy and use of these measures were lower following implementation. Nonetheless, we are encouraged that this method is promising and that the reporting of evidence-supported mental health treatment may be on track to becoming a routine feature of quality monitoring in children’s mental health.

## Data Availability

Data from this study can be accessed at Open Science Framework: https://osf.io/ct7k5/files/.

## Abbreviations

EST: Evidence-supported treatments
CMH: Community mental health
IOM: The Institute of Medicine
CHIP: Children’s Health Insurance Programs
MMIS: Washington State Health Care Authority Medicaid Management Information System
HCA: Health Care Authority
AIM: Acceptability of Intervention Measure
IAM: Intervention Appropriateness Measure
FIM: Feasibility of Intervention Measure
CPT: Current Procedural Terminology
MCO: Managed care organization
ANOVA: Analysis of variance
EHR: Electronic health record
IMC: Integrated Managed Care
SERI: Service Encounter Reporting Instructions
COE4CCN: Center of Excellence on Quality Care Measures for Children with Complex Needs
